# Cephalometric Floating Norms for the *β* Angle and MMBP-Wits

**DOI:** 10.1155/2018/8740731

**Published:** 2018-04-04

**Authors:** Giuseppe Perinetti, Michele Ceschi, Alessandro Scalia, Luca Contardo

**Affiliations:** ^1^Private Practice, Nocciano, Italy; ^2^Department of Medical, Surgical and Health Sciences, School of Dentistry, University of Trieste, Trieste, Italy

## Abstract

The importance of an accurate measurement of sagittal jaw relationship, that is, skeletal class, is critical to orthodontic treatment planning. The ANB angle, *β* angle, and MMBP-Wits are among indices of sagittal jaw relationship. All of these indices are subjected to geometrical distortion, especially from facial divergence, making the use of floating (individualized) norms necessary. This study thus provides floating norms for the ANB angle and for the first time for the *β* angle and MMBP-Wits. Lateral head films were obtained from 119 subjects (74 females and 45 males; mean age, 11.2 ± 1.5 years; range, 8.2–14.0 years) with well-balanced and pleasant profile and a near-ideal occlusion. Multiple regression models were employed to quantify the association of the ANB angle, *β* angle, and MMBP-Wits each with other four angular cephalometric parameters including SNA, SN/PP, SN/MP, and NSBa angles. The *β* angle and MMBP-Wits were associated with the SNA and SN/MP angles; the ANB angle was associated with all the four other cephalometric variables. Floating norms for the *β* angle and MMBP-Wits (but not ANB angle) have been cross-tabulated according to the SNA angle (from 74° to 90°) and SN/MP angle (from 24° to 44°). While the ANB angle is subjected to significantly more geometrical distortion as compared to the *β* angle and MMBP-Wits, floating norms may be used to individualize the reference values for both the *β* angle and MMBP-Wits.

## 1. Introduction

The importance of an accurate measurement of sagittal jaw relationship, that is, skeletal class, is critical to orthodontic treating planning [1–3]. In this regard, both angular and linear measurements have been incorporated into various cephalometric analyses to help the clinician to diagnose the skeletal class and establish the most appropriate treatment plan.

Among the most popular cephalometric indices of sagittal jaw relationship is the ANB angle [1, 2] for which, however, a difference between the interpretation of this angle and the actual jaw relationship has been reported [3–6]. A second widely used measurement, the Wits appraisal on the functional occlusal plane [3], was introduced to overcome problems related to the ANB angle. However, the Wits appraisal relates points A and B to the occlusal plane and it suffers limitations related to the identification and the cant of this plane [7]. To overcome these limitations, more indices have been developed recently including the *β* angle [8] and the maxillary-mandibular plane angle bisector (herein referred to as MMBP-Wits) [9]. According to the authors proposing these indices they would suffer less geometrical distortion from facial divergence or jaw rotation as compared to others [8, 9], even though these aspects have still to be investigated.

Another method to overcome geometrical distortion was introduced by Järvinen [10] through the floating norms of the ANB angle for different facial types, showing that this concept proves advantageous in practical orthodontics. However, all floating norms are mostly based on Steiner's analysis [11–14] and no studies regarding other indexes of sagittal jaw relationship have been performed. The aim of this study is to provide floating norms for the ANB angle, *β* angle, and MMBP-Wits according to facial divergence to obtain individualized cephalometric norms related to each facial type.

## 2. Materials and Methods

### 2.1. Study Population and Design

The database between January 2009 and August 2017 of the Sections of Stomatology of the Department of Medical, Surgical and Health Sciences, University of Trieste, was screened. This study included subjects who were seeking orthodontic treatment for minimal tooth alignment and who had never been treated before. As a routine procedure, signed informed consent for releasing diagnostic material for scientific purposes was obtained from the patients' parents prior to entry into treatment, and protocol was approved by the local Ethical Committee. In particular, a first clinical session, extra- and intraoral photographs, impressions, and a lateral cephalogram were taken as part of the pretreatment clinical recording. In order to be included in the study, subjects had to present with a well-balanced and pleasant profile and a near-ideal occlusion. The following inclusion criteria were applied: (i) age between 8 and 14 years; (ii) absence of ant craniofacial anomaly or extensive dental caries or restorations; (iii) good general health; (iv) no history of trauma at the craniofacial region; and (v) Caucasian ethnicity. A total of 119 subjects (74 females and 45 males) were included in the study (mean age, 11.2 ± 1.5 years; range, 8.2–14.0 years).

### 2.2. Clinical Recordings

An experienced orthodontist (MC) screened the cases for inclusion. Extraoral frontal and lateral photographs were analyzed along with the corresponding lateral head cephalogram to assess the subject profile. Intraoral photographs and stone models were used to assess a near-ideal occlusion. In particular, the subject had to have Class I molar relationship with less than half-cusp displacement and canine (either deciduous or permanent, where assessable) relationships with less than 1/4 of cusp displacement [15], normal overjet and overbite, and minimal incisor irregularities. Subjects with missing maxillary lateral incisors were included if the other conditions were met. A second experienced orthodontist (GP) analyzed the recordings to ensure correct enrollment, and discussion between operators was executed in case of disagreement.

### 2.3. Cephalometric Analysis

**Figure 1:**
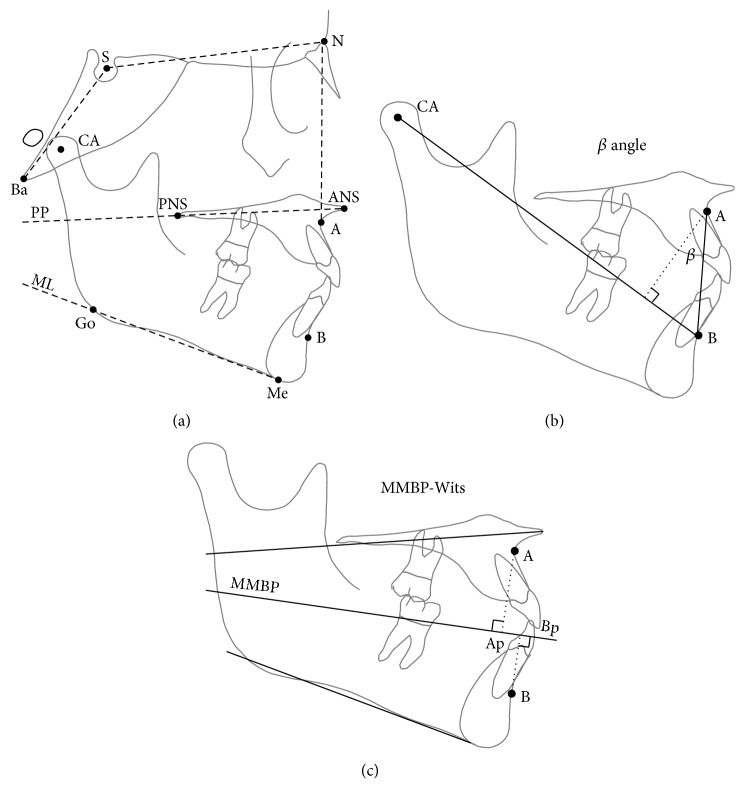
Cephalometric analyses used in the study.* Landmarks*. A, subspinale; B, supramentale; N, nasion; S, centre of the sella turcica; Ba, basion; ANS, anterior nasal spine; PNS, posterior nasal spine; Me, menton; Go, gonion; CA, axis of the condyle. Planes: PP, palatal plane; ML, mandibular plane. See text for details.

A customized digitization regimen and analysis with cephalometric software (Viewbox, version 3.0, dHAL Software, Kifissia, Greece) were used for all cephalograms examined in this study. The cephalometric analysis required the digitization of 10 landmarks (Figure 1(a)). Indices of sagittal jaw relationship were the ANB angle [1]; the *β* angle (Figure 1(b)) was defined as the angle between the perpendicular line from point A to the CA-B line and the A-B line [8], while the MMBP-Wits (Figure 1(c)) was defined as the distance between the perpendicular projection of A and B (Ap and Bp, resp.) on the bisector of the PP/MP angle [9]. Other cephalometric parameters included four angular measurements as follows (Figure 1(a)): maxillary prognathism (SNA angle), maxillary inclination relative to the cranial base (SN/PP angle), mandibular inclination relative to the cranial base (SN/MP angle), and cranial base angle (NSBa angle). Lateral cephalograms were standardized as to real dimensions, that is, magnification factor of 0%. All sets of cephalograms were traced by an expert orthodontist (MC) and a second investigator (LC) checked each tracing for accuracy.

### 2.4. Method Error and Statistical Analysis

With the aim of quantifying the full method error of the recordings for either palatal parameter, the method of moments (MME) variance estimator was used [16]. Therefore, (MME) variance estimator was calculated for each cephalometric variable on a pair of 20 repeated recordings randomly selected.

The SPSS software (SPSS® Inc., Chicago, Illinois, USA) was used to perform the subsequent data analysis. Descriptive statistics for each investigated parameter included mean, standard deviation (SD), median, minimum, and maximum. Moreover, associations between SNA, SN/PP, SN/MP, and NSBa angles (explanatory variables) with each ANB angle, *β* angle, and MMBP-Wits (dependent variables) were evaluated by backward multiple linear regresison models. The cut-off levels of significance used were 0.01 and 0.05 for entry and removal, respectively. Moreover, for the *β* angle and MMBP-Wits the final models, that is, regression equations, were used to calculate the floating norms according to the significantly independent variables as previously reported [10].

A *p* value < 0.05 was considered statistically significant.

## 3. Results

The errors for angular measurements ranged from 0.5° (ANB angle) to 1.2° (SN/MP angle). Errors for the MMBP-Wits were of 0.6 mm. Descriptive statistics for each analyzed parameter is reported in Table 1.

Results on the backward multiple linear regressions are summarized in Table 2. All the three cephalometric parameters of anteroposterior maxillomandibular relationship had significant association with the explanatory variables with *R*^2^ ranging from 0.183 to 0.212 for the MMBP-Wits and ANB angle, respectively. The ANB angle was significantly associated with all the explanatory variables. In particular, the SNA, SN/MP and NSBa angles were positively associated with the ANB angle, while the SN/PP angle was inversely associated. On the contrary, both the *β* angle and MMBP-Wits were significantly associated with SNA and SN/MP angles. These explanatory variables showed positive and inverse association with the *β* angle and MMBP-Wits, respectively.

Regression equations were thus derived for the *β* angle and MMBP-Wits as follows: *β* angle = 0.438 · SNA angle + 0.274 · SN/MP angle − 13.525, and MMBP-Wits = −0.321 · SNA angle − 0.219 · SN/MP angle + 30.743. According to these equations, floating norms were derived for each parameter according to the variations of SNA and SN/MP angles as reported in Table 3.

## 4. Discussion

Through the use of multivariate models, floating norms for two of the three indexes of sagittal jaw relationship have been provided for Caucasian subjects. Among the investigated cephalometric parameters potentially distorting the indices of jaw relationship, the SNA angle and SN/MP angle were the most important.

With the aim of identifying a reliable index of sagittal jaw relationship, various analyses have been introduced in literature such as AXB angle [5], Pi analysis [17], and W angle [18]. However, these indexes (including the others herein investigated) are based on geometric relationships and anatomical landmarks and they can be influenced especially by facial divergence and different morphological localization of these landmarks [3, 5, 6, 19, 20].

All of these parameters are based on standard mean values which are determined from populations subjects with an ideal occlusion and well-balanced faces. Solow [21] showed that a certain pattern may be correlated with cephalometric skeletal variables. This means that even though all the cephalometric variables of a patient lie at or beyond one standard deviation they might be accepted if they only have a certain relation to each other. Moreover, these indices are geometrically sensitive and can give false results. In particular, a morphological feature that has been shown to affect dramatically the reliability of cephalometric indices of skeletal class is the facial divergence [5, 6]. However, most of the published literature investigating these distorting effects has focused on the ANB angle and FOP-Wits [4, 6, 7, 19, 22, 23] with most of the other indices still needing further investigations to describe if and how morphological variables, such as facial divergence, affect their reliability in terms of sagittal relationship of the jaws. In particular, for the *β* angle [8] and MMBP-Wits [9], validations have been reported only from the proposing authors. Further limitations of previous studies reside in the concept that either only subjects showing a normal divergence were analyzed [8], or a bivariate correlations analysis has been used to investigate the agreement between two cephalometric indices of skeletal class [19].

The results regarding normal values for the ANB angle (2.9° ± 1.3) and *β* angle (30.3° ± 3.5) obtained in the present sample (Table 1) for skeletal Class I subjects are very similar to others previously reported [2, 8]. On the contrary, normal values for the MMBP-Wits seen herein were of −2.4 mm ± 2.4, while mean normal value has been reported to be −4 mm [9]. However, this difference would have little clinical relevance, and difference in the sample under investigation may be an explanation.

In the present study, the grade of correlation between angles SNA, SN/PP, SN/MP, and NSBa and with indices of sagittal jaw relationship (ANB, *β* angle, and MMBP-Wits) has been calculated (Table 2). Being not affected by magnification, angular parameters have been chosen for the analyses (with the exception of the MMBP-Wits). Thus, corresponding results would be more reproducible. Moreover, a large sample size has also been included. However, other cephalometric parameters, not tested heroin, might also be significantly responsive of potential geometrical distortion.

As the results showed, the ANB angle was the most affected parameter. In particular, even the NSBa angle may have an effect of distortion of this index of sagittal jaw relationship (Table 2). This evidence is in line with previous reports [4–6, 22, 23] and suggests that the ANB angle should not be used in patients showing noteworthy deviation from the norm, in terms of facial divergence or maxillary protrusion. On the contrary, the *β* angle and MMBP-Wits were showed to be less influenced by the analyzed cephalometric parameters, and hence intuitive floating norms could be derived (Table 3).

Using the ANB angle as a dependent variable and SNA, SN/PP, SN/MP, and NSBa as independent variables, resulting *R*^2^ is equal to 0.212; that is, all four cephalometric parameters influence ANB angle variability for a total of 21.2% of the value. *R*^2^ for the *β* angle and MMBP-Wits was 0.189 and 0.183, respectively. Therefore, the SNA angle and SN/MP angle alone would account for about 18-19% of their variability (Table 2).

Accordingly, regression equations for *β* angle and MMBP-Wits were as follows: *β* angle = 0.438 · SNA angle + 0.274 · SN/MP angle − 13.525 and MMBP-Wits = −0.321 · SNA angle − 0.219 · SN/MP angle + 30.743. With the use of such equations, floating norms have been obtained for most common situations of SNA angle (from 74° to 90°) and SN/MP angle (from 24° to 44°) (Table 3). With such floating norms, it is possible to analyze the sagittal jaw relationship by adjusting the *β* angle and MMBP-Wits for the maxillary protrusion and facial divergence.

Cephalometric diagnosis is based on the use of separate normal values resulting from a statistical population but the discovery of an influence on indexes of anteroposterior sagittal discrepancy, deriving from divergence, made this concept obsolete. Some subjects can have a first skeletal class relationship and a harmonic profile even distancing from norm values. Therefore, it is useful to replace cephalometric normal values with mean values deriving from a population sample with individual (floating) norms based on the association between appropriate cephalometric variables [10, 11, 14]. However, as floating norms are derived from regression equations, the accuracy of the diagnosis would depend on the standard errors retrieved in the equations.

According to the degree of maxillary protrusion (SNA angle) and facial divergence (SN/MP angle), clinicians may choose the less biased index of sagittal jaw relationship to achieve a more accurate diagnosis. Indeed, using the floating norms for SN/MP and SNA angles (Table 3) it is possible to derive individualized values for each patient. Moreover, considering the standard deviations herein derived (Table 1), it is possible to obtain the corresponding intervals of such individualized norms. If the value belongs to the interval given by mean value and standard deviation, the subject can be diagnosed to have normal sagittal jaw relationship, that is, skeletal Class I occlusion. Values outside such intervals would thus be indicative of skeletal Class II or Class III malocclusion.

## 5. Conclusions

- The ANB angle is subjected to significantly more geometrical distortion as compared to the *β* angle and MMBP-Wits.
- Floating norms have been provided to individualize the reference values for both the *β* angle and MMBP-Wits.

## Figures and Tables

**Table 1:** Descriptive statistics for the indexes of sagittal jaw relationship and other cephalometric parameters (*n* = 119).

Parameter	Mean ± SD	Median	Minimum	Maximum
Maxillomandibular, anteroposterior				
ANB (°)	2.9 ± 1.3	3.0	0.0	5.0
*β* angle (°)	30.3 ± 3.5	30.3	21.8	39.9
MMBP-Wits (mm)	−2.4 ± 2.4	−2.0	−9.4	3.7
Maxillomandibular and cranial base, anteroposterior and vertical				
SNA angle (°)	81.2 ± 3.3	80.7	72.2	89.8
SN/PP angle (°)	8.5 ± 15.7	7.0	0.7	174.6
SN/MP angle (°)	32.1 ± 4.6	31.6	21.5	44.6
NSBa angle (°)	129.4 ± 5.2	129.5	119.1	140.7

SD, standard deviation.

**Table 2:** Results of the backward multiple linear regressions for the indexes of sagittal jaw relationship with each explanatory variable (*n* = 119).

Explanatory variable	*β* (SE)	*t*
*Model 1: dependent variable ANB angle, R* ^2^ = 0.212		
SNA angle	0.175 (0.040)	4.330^*∗∗*^
SN/PP angle	−0.017 (0.007)	2.384^*∗*^
SN/MP angle	0.107 (0.027)	3.886^*∗∗*^
NSBa angle	0.056 (0.023)	2.392^*∗*^
*Model 2: dependent variable β angle, R* ^2^ = 0.189		
SNA angle	0.438 (0.101)	4.316^*∗∗*^
SN/MP angle	0.274 (0.074)	3.719^*∗∗*^
*Model 3: dependent variable MMBP-Wits, R* ^2^ = 0.183		
SNA angle	−0.321 (0.071)	−4.534^*∗∗*^
SN/MP angle	−0.219 (0.051)	−4.266^*∗∗*^

Results of the multiple linear regressions are presented as *β* coefficient (standard error); *R*^2^, coefficient of determination. Level of significance: ^*∗*^*p* < 0.05; ^*∗∗*^*p* < 0.001.

**Table 3:** Floating norms for the *β* angle and MMBP-Wits according to the SNA and SN/MP angles (*n* = 119).

Parameter	SN/MP angle (°)	SNA angle (°)								
		74	76	78	80	82	84	86	88	90
*β* angle (°)	24	25	26	27	28	29	30	31	32	32
	28	27	27	28	29	30	31	32	33	34
	32	28	29	29	30	31	32	33	34	35
	36	29	30	31	31	32	33	34	35	36
	40	30	31	32	32	33	34	35	36	37
	44	31	32	33	34	34	35	36	37	38
MMBP-Wits (mm)	24	2	1	0	0	−1	−1	−2	−3	−3
	28	1	0	0	−1	−2	−2	−3	−4	−4
	32	0	−1	−1	−2	−3	−3	−4	−5	−5
	36	−1	−2	−2	−3	−3	−4	−5	−5	−6
	40	−2	−2	−3	−4	−4	−5	−6	−6	−7
	44	−3	−3	−4	−5	−5	−6	−6	−7	−8

## Data Availability

Data used in the study is available upon request to the corresponding author.

## References

[1] Riedel R. A. (1952). The relation of maxillary structures to cranium in malocclusion and in normal occlusion. *The Angle Orthodontist*.

[2] Steiner C. C. (1953). Cephalometrics for you and me. *American Journal of Orthodontics and Dentofacial Orthopedics*.

[3] Jacobson A. (1975). The “Wits” appraisal of jaw disharmony. *American Journal of Orthodontics and Dentofacial Orthopedics*.

[4] Järvinen S. (1985). An analysis of the variation of the ANB angle: A statistical appraisal. *American Journal of Orthodontics and Dentofacial Orthopedics*.

[5] Freeman R. S. (1981). Adjusting A-N-B angles to reflect the effect of maxillary position. *The Angle Orthodontist*.

[6] Hussels W., Nanda R. S. (1987). Clinical application of a method to correct angle ANB for geometric effects. *American Journal of Orthodontics and Dentofacial Orthopedics*.

[7] Rushton R., Cohen A. M., Linney A. D. (1991). The relationship and reproducibility of angle ANB and the Wits appraisal.. *British Journal of Orthodontics*.

[8] Baik C. Y., Ververidou M. (2004). A new approach of assessing sagittal discrepancies: The Beta angle. *American Journal of Orthodontics and Dentofacial Orthopedics*.

[9] Hall-Scott J. (1994). The maxillary-mandibular planes angle (MMo) bisector: A new reference plane for anteroposterior measurement of the dental bases. *American Journal of Orthodontics and Dentofacial Orthopedics*.

[10] Järvinen S. (1986). Floating norms for the ANB angle as guidance for clinical considerations. *American Journal of Orthodontics and Dentofacial Orthopedics*.

[11] Franchi L., Baccetti T., McNamara J. A. (1998). Cephalometric floating norms for North American adults. *The Angle Orthodontist*.

[12] Hasund A., Böe O. E. (1980). Floating norms as guidance for the position of the lower incisors.. *The Angle Orthodontist*.

[13] Segner D. (1989). Floating norms as a means to describe individual skeletal patterns. *European Journal of Orthodontics*.

[14] Tollaro I., Baccetti T., Franchi L. (1996). Floating norms for the assessment of craniofacial pattern in the deciduous dentition.. *European Journal of Orthodontics*.

[15] Perinetti G., Cordella C., Pellegrini F., Esposito P. (2008). The prevalence of malocclusal traits and their correlations in mixed dentition children: results from the Italian OHSAR Survey. *Oral Health & Preventive Dentistry*.

[16] Perinetti G. (2016). StaTips Part II: Assessment of the repeatability of measurements for continuous data. *South European Journal of Orthodontics and Dentofacial Research*.

[17] Kumar S., Valiathan A., Gautam P., Chakravarthy K., Jayaswal P. (2012). Anevaluation o. The Pi analysis i. The assessment of anteroposterior jaw relationship. *Journal of Orthodontics*.

[18] Bhad W. A., Nayak S., Doshi U. H. (2013). A.new approach of assessing sagittal dysplasia: The W.angle. *European Journal of Orthodontics*.

[19] Oktay H. (1991). A comparison of ANB, WITS, AF-BF, and APDI measurements. *American Journal of Orthodontics and Dentofacial Orthopedics*.

[20] Taylor C. M. (1969). Changes in the relationship o f nasion, point A, and point B and the effect upon ANB. *American Journal of Orthodontics and Dentofacial Orthopedics*.

[21] Solow B. (1996). The pattern of craniofacial associations - a morphological and methodological correlation and factor analysis study on young male adults. *Acta Odontologica Scandinavica*.

[22] Hurmerinta K., Rahkamo A., Haavikko K. (1997). Comparison between cephalometric classification methods for sagittal jaw relationships. *European Journal of Oral Sciences*.

[23] Rotberg S., Fried N., Kane J., Shapiro E. (1980). Predicting the "Wits" appraisal from the ANB angle. *American Journal of Orthodontics and Dentofacial Orthopedics*.

